# Function of the endolysosomal network in cholesterol homeostasis and metabolic-associated fatty liver disease (MAFLD)

**DOI:** 10.1016/j.molmet.2020.101146

**Published:** 2021-01-05

**Authors:** Dyonne Y. Vos, Bart van de Sluis

**Affiliations:** Department of Pediatrics, section Molecular Genetics, University of Groningen, University Medical Center Groningen, Groningen, the Netherlands

**Keywords:** NAFLD, Endosome and lysosome, Cholesterol transport, Endosomal sorting, LDLR, PCSK9

## Abstract

**Background:**

Metabolic-associated fatty liver disease (MAFLD), also known as non-alcoholic fatty liver disease, has become the leading cause of chronic liver disease worldwide. In addition to hepatic accumulation of triglycerides, dysregulated cholesterol metabolism is an important contributor to the pathogenesis of MAFLD. Maintenance of cholesterol homeostasis is highly dependent on cellular cholesterol uptake and, subsequently, cholesterol transport to other membrane compartments, such as the endoplasmic reticulum (ER).

**Scope of review:**

The endolysosomal network is key for regulating cellular homeostasis and adaptation, and emerging evidence has shown that the endolysosomal network is crucial to maintain metabolic homeostasis. In this review, we will summarize our current understanding of the role of the endolysosomal network in cholesterol homeostasis and its implications in MAFLD pathogenesis.

**Major conclusions:**

Although multiple endolysosomal proteins have been identified in the regulation of cholesterol uptake, intracellular transport, and degradation, their physiological role is incompletely understood. Further research should elucidate their role in controlling metabolic homeostasis and development of fatty liver disease.

## Highlights

• The intracellular cholesterol transport is tightly regulated by the endocytic and lysosomal network.
• Dysfunction of the endolysosomal network affects hepatic lipid homeostasis.
• The endosomal sorting of lipoprotein receptors is precisely regulated and is not a bulk process.

## 1 Introduction

Metabolic-associated fatty liver disease (MAFLD, previously known as non-alcoholic fatty liver disease, NAFLD) [1] – the most common cause of chronic liver disease worldwide – refers to a range of liver disorders, but the hallmark of MAFLD is lipid accumulation in the liver (simple steatosis) [2]. Its global prevalence was estimated at 25% in 2018 [2]. The majority of patients have simple steatosis, but approximately 25% of MAFL patients show signs of liver inflammation (steatohepatitis). Although this inflammatory injury is reversible, it can progress to fibrosis, cirrhosis, and hepatocellular carcinoma [3]. The development of MAFLD is often accompanied by features of metabolic syndrome, such as type 2 diabetes, central obesity, elevated plasma triglycerides (TGs), hypertension, and decreased high-density lipoprotein (HDL) cholesterol [2,4].

Excessive accumulation of hepatic TGs can be the result of disturbed balance in the flux of dietary and adipose tissue-derived free fatty acids (FFAs) to the liver, hepatic *de novo* lipogenesis (DNL), and mitochondrial β-oxidation of FFAs [3,5]. Studies have also demonstrated that dysregulation of cholesterol metabolism contributes to the pathogenesis of MAFLD [6]. Cholesterol homeostasis is maintained through intracellular cholesterol-sensing machineries and transcription factors that regulate cholesterol synthesis, uptake, and excretion. Disruption of these pathways in MAFLD leads to cholesterol toxicity and lipid accumulation [7].

An essential aspect in the maintenance of cholesterol homeostasis is the intracellular transport of cholesterol. After cholesterol is endocytosed, it is transported through the endolysosomal network, an interconnected network of membranous compartments, which enables the subsequent distribution of cholesterol to other cellular organelles [8,9]. Various studies have demonstrated that impaired functioning of the endolysosomal system results in dysregulated glucose and lipid homeostasis [[10], [11], [12], [13], [14], [15]], which indicates that the endolysosomal network has an important role in metabolic regulation, including cholesterol metabolism.

Although most cholesterol transport pathways through subcellular compartments have been well-described [16,17], their mechanisms and regulation remain incompletely understood. This review provides an overview of our current knowledge of the tight regulation of the endolysosomal network and its role in cholesterol homeostasis and in the pathogenesis of MAFLD.

## 2 The endolysosomal network controls cellular cholesterol uptake and intracellular cholesterol transport

**Figure 1:**
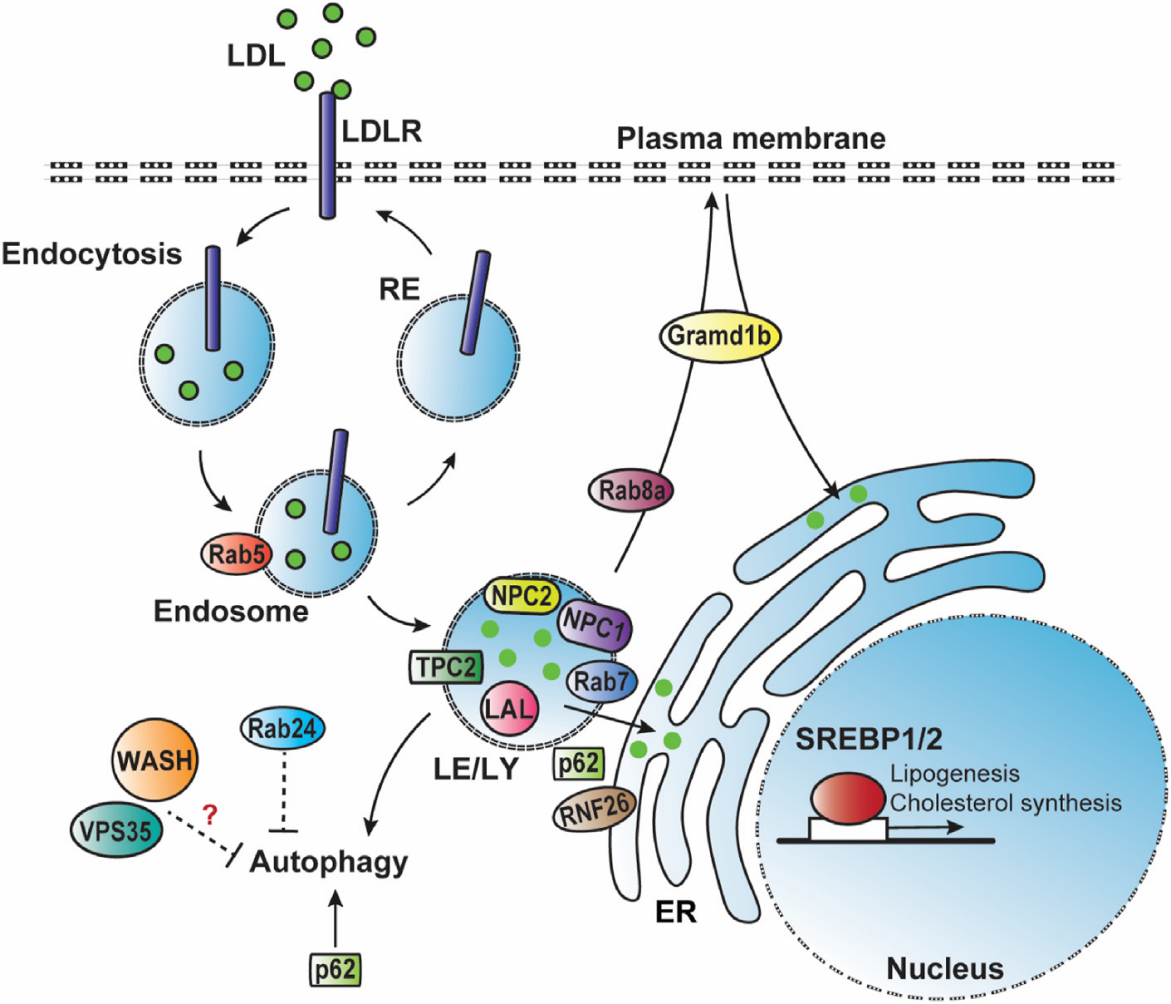
**Cholesterol transport through the endolysosomal network.** After binding to LDLR, LDL-cholesterol is internalized, and the LDL-LDLR complex is transported to endosomes, where LDL-cholesterol dissociates from LDLR. LDLR can be transported back to the plasma membrane via recycling endosomes (RE). LDL-cholesterol is transported to late endosome/lysosome (LE/LY), where lysosomal acid lipase (LAL) hydrolyzes cholesterol esters to release free cholesterol. TPC2 is important for LE-LY fusion and thus for further cholesterol transport. NPC2 can bind and deliver free cholesterol to NPC1. NPC1 regulates cholesterol transport to other cellular compartments, such as the endoplasmic reticulum (ER) by interacting with Rab7 and the plasma membrane via Rab8a. The ER-located RNF26 recruits and ubiquitinates p62, leading to the binding of specific endosomal-associated adaptor proteins to mediate the positioning of vesicles in the perinuclear area, but its function in cholesterol transport needs to be elucidated. Gramd1b, or Aster-B, is implicated in both LE/LY-to-PM and PM-to-ER cholesterol transport. When cholesterol levels in the ER are low, SREBP is translocated to the nucleus, where it induces transcription of genes involved in lipogenesis and cholesterol uptake and synthesis. Cholesterol levels in LE/LY affect lysosomal function and thereby also the autophagic pathway, in which p62 is an important player. Rab24 is thought to reduce autophagy and thereby contribute to MAFLD. The endosomal WASH complex and the retromer subunit VPS35 are also known to affect autophagy, but their physiological role in this context and in MAFLD remain unknown.

The endolysosomal network is crucial for the cell to adapt to intracellular and environmental changes. As it regulates the internalization and sorting of a wide range of integral proteins, such as signaling receptors, lysosomal hydrolase receptors, adhesion molecules, and nutrient transporters, it is hence vital for maintaining cellular homeostasis [9]. The cellular uptake of the macromolecule, cholesterol, is highly dependent on receptor-mediated endocytosis (Figure 1). Cholesterol that enters hepatocytes originates mainly from cholesterol-rich low-density lipoprotein (LDL) particles. LDL binds to the LDL receptor (LDLR) at the cell surface, and together they are internalized via clathrin-mediated endocytosis and further transported to early endosomes [18]. Acidification of the endosomal lumen induces dissociation of LDL-cholesterol from LDLR, after which the receptor is either recycled back to the plasma membrane (PM) or directed to the lysosomes for degradation [19] (see also Section 6). LDL-cholesterol is transported to late endosomes and lysosomes (LE/LY), where cholesteryl esters (CEs) are hydrolyzed by acid lipases to release free cholesterol. The Niemann-Pick type C2 (NPC2), a small luminal protein, binds to free cholesterol and subsequently delivers it to Niemann-Pick type C1 (NPC1). NPC1 is a large transmembrane protein localized on the limiting membrane of LE/LY [20] (Figure 1). From here, cholesterol is distributed to other cellular compartments [8,9]. The importance of NPC1 and NPC2 in cholesterol transport is illustrated in patients with NPC disease, a severe neurodegenerative disorder caused by loss-of-function mutations in *NPC1* or *NPC2*. Dysfunction of these proteins results in accumulation of cholesterol in LE/LY compartments, leading mainly to neurological symptoms, but also to progressive liver disease characterized by cholestasis, hepatomegaly, and fibrosis or cirrhosis [[21], [22], [23]]. A genome-wide association study identified a single-nucleotide polymorphism (SNP) in *NPC1* associated with obesity [24], and NPC1 haploinsufficiency in mice induces hepatosteatosis and features of metabolic syndrome [25]. These findings indicate that NPC1 may play additional roles in metabolic homeostasis other than transporting cholesterol from the LE/LY.

Previously, the membrane protein TMEM97 has been identified as an NPC1-interacting protein and regulator of cellular cholesterol levels [26]. Decreased TMEM97 levels result in enhanced NPC1 protein levels, along with restored cholesterol transport and decreased lipid accumulation in NPC *in vitro* models [27]. Despite these significant findings, the authors could not substantiate the *in vitro* results in hepatocytes of a murine NPC model, which indicates that the effect of TMEM97 on NPC1 functioning might be cell type specific [27]. Another study has shown that TMEM97 may also be involved in LDL cholesterol uptake through a direct interaction with LDLR [28]; thus, further research is required to understand the contribution of TMEM97 in NPC disease and hepatic cholesterol uptake.

The importance of the endolysosomal network in cholesterol homeostasis and fatty liver disease is further supported by a study showing that impaired fusion of endolysosomal compartments induces defects in LDL cholesterol transport and degradation [29]. Upon activation, the two-pore channel (TPC) 2, a cation channel located mainly in the membrane of LE/LY compartments, releases Ca2+ to regulate endolysosomal fusion processes (Figure 1). Where LE-LY fusion might normally be induced by Ca2+ released from TPC2 channels, deficiency of TPC2 impairs this process and results in cholesterol accumulation in a late endosomal compartment. TPC2-deficient mice fed a cholesterol-rich diet are prone to develop steatosis, accompanied by hypercholesterolemia and signs of liver damage [29].

## 3 Cholesterol transport to the endoplasmic reticulum (ER) is necessary to maintain cholesterol homeostasis

After export from LE/LY compartments, cholesterol is transported to other cellular compartments, such as the plasma membrane (PM), mitochondria, and ER [8,9]. The ER has a key role in intracellular cholesterol sensing and regulation. Here, cholesterol controls the processing of sterol regulatory-element binding proteins (SREBPs), a family of transcription factors regulating cellular lipid homeostasis (reviewed in [30,31]) (Figure 1). Briefly, by binding to two chaperones, SREBP-cleavage activating protein (SCAP) and insulin-induced gene protein 1 (INSIG1), sterols prevent the translocation of SREBP to the Golgi complex [32,33]. Under sterol-poor conditions, the SCAP-SREBP complex is no longer retained in the ER and is trafficked to the Golgi. Here, SREBP is processed to its mature form, which enters the nucleus to activate the transcription of genes involved in cholesterol synthesis and uptake (mainly by the SREBP-2 isoform) and lipogenesis (mainly by the SREBP-1c isoform) [34].

Studies in patients have indicated that MAFLD is associated with upregulated SREBP expression [35,36], and data from mouse models demonstrate that increased activation of SREBP is a causal factor in the development and progression of fatty liver disease [[37], [38], [39], [40]]. The observation that loss of SREBP-1 upregulates SREBP-2, while SREBP-1 activity depends on functional SREBP-2 signaling [34,41,42], indicates that there is a tight crosstalk and differential regulation of two isoforms. Although several regulators in governing of SREBP signaling have been identified [34], the underlying mechanisms are not fully understood and are currently still being elucidated. Recently, four independent groups have identified C12orf49 as a novel factor that controls SREBP signaling [[43], [44], [45], [46]]. Loregger et al. showed that C12orf49 (dubbed SPRING) controls SREBP signaling by regulating the localization and levels of SCAP [43]. Data from Xiao et al. and Bayraktar et al. indicate that C12orf49 (renamed POST1) mediates SREBP activity through an interaction with site-1 protease (S1P), which cleaves SREBP at the Golgi, followed by a second cleavage of SREBP by site-2 protease (S2P) [44,45,47]. According to Xiao et al., C12orf49 (POST1) promotes formation of the mature form of S1P, subsequently facilitating the translocation of mature S1P from the ER to the Golgi [44]. Thus, the SCAP-SREBP axis to sense intracellular cholesterol content is tightly regulated to maintain lipid homeostasis and prevent hepatic fat accumulation.

## 4 Endolysosomal proteins are important regulators of cholesterol transport to the ER

The exact routes of cholesterol transport from LE/LY to the ER are not clearly defined. Over the past decades, studies have provided evidence that cholesterol released from LE/LY first reaches the PM, prior to distribution of excess cholesterol to the ER [[48], [49], [50], [51]] (Figure 1). However, other studies have shown that cholesterol can also travel directly from LE/LY to the ER [[52], [53], [54], [55]]. It has been estimated that approximately 30% of LDL-derived cholesterol follows this direct pathway, and a study has shown that cyclodextrin, an extracellular acceptor of cholesterol from the PM, blunted only 70% of cholesterol esterification in the ER [52].

Membrane contact sites (MCS), zones where different organelle membranes come into close apposition (10–30 nm) to each other without fusion, have been shown to be sites of non-vesicular cholesterol transfer between organelles [56]. Lipid transfer proteins are required to facilitate this transport by cross-bridging MCS (reviewed in [16]). As such, oxysterol-binding protein-related protein 1L (ORP1L), ORP5, and StAR-related lipid transfer domain containing 3 (StARD3) are implicated in direct LE/LY-to-ER cholesterol transport [54,57,58]. Also, the late endosomal protein Rab7 is involved in the formation of LE/LY-ER MCS related to lipid transfer (see section 5). Moreover, a recent study has identified an additional role for NPC1 in the formation of LE/LY-ER MCS by interacting with the ER-resident sterol-binding protein Gramd1b to facilitate cholesterol egress from LE/LY [55].

**Figure 2:**
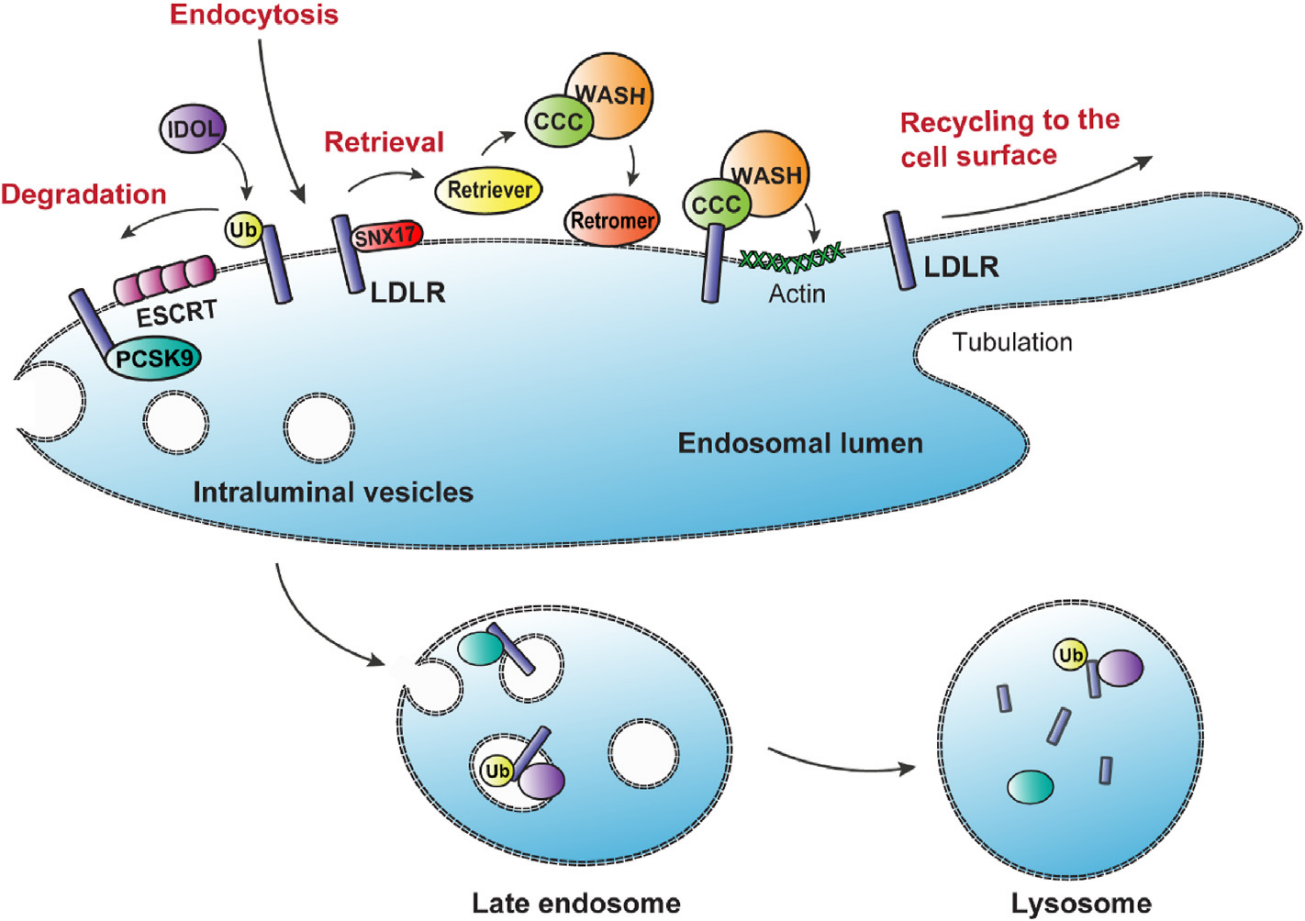
**Working model of endosomal trafficking of LDLR.** Following endocytosis, LDLR is transported to endosomes, where the receptor is directed to the late endosomes and lysosomes for PCSK9- or IDOL-mediated degradation or is retrieved from this pathway and recycled back to the plasma membrane. The CCC complex is known to facilitate recycling of LDLR by a direct interaction between CCC subunit COMMD1 with LDLR, whereas WASH drives formation of actin patches on endosomal sorting domains to regulate LDLR recycling. Although the function of retromer and retriever in LDLR recycling has not been fully elucidated, retromer might facilitate LDLR recycling by recruiting CCC and WASH complexes to the endosomal membrane, and retriever might regulate LDLR retrieval and recycling by coupling to SNX17, an adaptor protein binding to the NPxY-motif in the cytosolic tail of LDLR. ESCRT = endosomal sorting complex required for transport; Ub = ubiquitin.

Another interesting player in LE/LY-to-ER cholesterol transport might be p62/SQSTM1. Although mostly known for its role in autophagy [59] (see also section 7), it was recently shown that p62 participates in a molecular bridge to position related vesicles (e.g., recycling early and late endosomes) in the perinuclear region in an autophagy-independent manner (Figure 2) [60]. Following ubiquitination of p62 by the ER-localized RNF26, endosomal vesicles are recruited to dock at the ER membrane to enable dynamic cargo trafficking [60]. However, it remains unclear whether p62 plays a significant role in cholesterol transport from the endosomal vesicles to the ER and, subsequently, in metabolic diseases, independent of its function in autophagy (section 7).

Although most cholesterol is directly transported to the PM, the mechanisms underlying these transport pathways are not well characterized. One potential pathway is the Rab8a-dependent transport of cholesterol-enriched LE/LY-derived vesicles to the PM (Figure 1). After recruitment of Rab8a to LE/LY by NPC1, cholesterol-enriched vesicles are transported along cortical actin toward the PM, where cholesterol delivery occurs at focal adhesion sites [61]. Interestingly, there is no fusion between the vesicles and PM, suggesting that this pathway might rely on an unidentified MCS [17,61]. In contrast, another potential transport route is associated with LE-PM fusion. Here, the ER-resident protein protrudin interacts with late endosomal Rab7 and phosphatidylinositol 3-phosphate to form ER-LE contacts, and subsequently binds the kinesin-1 motor protein to facilitate plus-end transport of LEs to the PM [62]. To date, it has not yet been determined whether this pathway is linked to cholesterol transfer.

Gramd1b, also known as Aster-B, has also recently been implicated in PM-to-ER cholesterol transport [63,64]. This pathway mediates the levels of accessible cholesterol in the PM and thus also the cholesterol levels in the ER, thereby regulating the activity of SREBP signaling [65,66]. The Aster-mediated PM-to-ER cholesterol transport was recently presented as a mechanistic link between increased hepatocyte cholesterol levels in steatosis and progression to fibrotic nonalcoholic steatohepatitis (NASH) through regulation of TAZ [67].

## 5 Rab GTPases are required for intracellular trafficking of lipids

The importance of the endolysosomal system in metabolism and MAFLD has also been illustrated in different mouse studies on the Rab family of small GTPases. In humans, the Rab family consists of more than 60 members and is known to regulate intracellular membrane traffic between organelles. Specific Rabs are localized on each organelle and associated vesicles, where they act as molecular ‘on/off’ switches to interact with effectors and to regulate the assembly of protein machinery [68].

Rab5, a Rab protein localized to early endosomes (EE), is thought to be required for the biogenesis of the endolysosomal system *in vivo* and is downregulated in NASH patients [69,70]. Hepatic knockdown of *Rab5* was accompanied by severe changes in both glucose and lipid metabolism, including impaired LDL cholesterol uptake, which resulted in increased steroid biosynthesis, hypercholesterolemia, and increased hepatic cholesterol and CE levels [10].

Remarkably, it has been demonstrated that the endosomal Rab5 is also associated with lipid droplets (LDs), lipid-storing organelles that are accumulated in MAFLD. By recruiting early endosomal antigen 1 (EEA1), Rab5 could promote contact between EE and LD [71], but whether these contacts have functional relevance in lipid transfer remains to be elucidated.

In contrast to Rab5, Rab24 is upregulated in livers of obese patients and patients with MAFLD, and this was found to be positively correlated with body fat [72]. Rab24 has been initially identified as a protein localized to LE, ER, and cis-Golgi regions [73,74]. However, in the recent study, knockdown of *Rab24* inhibited mitochondrial fission, resulting in enhanced mitochondrial respiration. This was associated with increased autophagic flux [72]. Mechanistically, Rab24 was demonstrated to directly bind the mitochondrial protein FIS1, thereby regulating the assembly of protein machinery required for mitochondrial fission. The upregulation of Rab24 in MAFLD might point to reduced autophagy and decreased mitochondrial respiration, both of which contribute to increased energy storage [72].

The late endosomal protein Rab7, one of the better characterized Rab proteins, is considered a major regulator of late endosomal transport and maturation [75]. In addition, multiple studies have indicated that Rab7 is an important player in the formation of LE/LY-ER MCS to facilitate cholesterol transfer (Figure 1). By interacting with PDZD8 at the ER membrane, Rab7 forms contacts with the ER where lipid transport might occur [76]. Another identified pathway involves AnnexinA6, which inactivates Rab7 by binding the Rab7-GTPase-activating protein TBC1D15 [58]. When AnnexinA6 and TBC1D15 are depleted in NPC1 mutant cells, Rab7 is active, and this is associated with increased StARD3-dependent LE-ER MCS formation, resulting in reduced cholesterol accumulation in LEs. These findings show that in cases of AnnexinA6 deficiency, Rab7 could facilitate cholesterol transfer via LE-ER MCS for storage in LDs [58]. In line with this study, recent findings have demonstrated that cells with inactive Rab7 show impaired intracellular LDL transport and free cholesterol accumulation in LE/LY, whereas Rab7 overexpression in fibroblasts of an NPC patient rescued LE/LY cholesterol accumulation [77]. In addition, active Rab7 was found to interact with NPC1, indicating that Rab7 might be a regulator of NPC1 [77]. Although *in vivo* evidence is still lacking, these studies illustrate that Rab7 plays a key role in cholesterol egress from the endolysosomal system. In addition to its localization to endolysosomal compartments, Rab7 is also found on autophagosomes. Here, Rab7 is involved in multiple autophagic processes (reviewed in [75]), including the regulation of lipid degradation (‘lipophagy’), by driving fusion between LE/LY and autophagic LDs [78].

## 6 The endosomal sorting machinery is essential for hepatic cholesterol uptake

Cholesterol that is taken up by the liver is derived mainly from plasma LDL cholesterol, which is internalized through LDLR [79], a mechanism that was described for the first time in the 1980s by Goldstein and Brown. Mutations in *LDLR* cause familial hypercholesterolemia (FH) [80], an autosomal dominant disorder characterized by very high plasma LDL cholesterol levels and increased risk of premature atherosclerotic cardiovascular disease [81]. Although mutations in *LDLR* are the most prevalent causal mutations among FH patients, mutations in genes regulating LDLR endocytosis, trafficking, and degradation are also associated with FH (reviewed in [82,83]). Identification of these genes has been crucial for the current molecular understanding of the intracellular LDLR trafficking pathway and the hepatic cholesterol uptake machinery [82].

After internalization of the LDL-LDLR complex and arrival at the endosomes, LDLR can be sorted toward the lysosomes for degradation or be directed back to the PM for reuse (Figure 2). Degradation of the receptor is coordinated by two distinct pathways (Figure 2). The inducible degrader of the LDL receptor (IDOL) ubiquitinates LDLR intracellularly, thereby tagging it for degradation, while proprotein convertase subtilisin/kexin type 9 (PCSK9) regulates LDLR proteolysis by binding to the receptor either intracellularly or extracellularly [[84], [85], [86], [87]]. Intracellularly, PCSK9 and LDLR can interact within the secretory pathway, and this leads to direct trafficking of the receptor from the Golgi network to the LE/LY for degradation [87]. Extracellular binding of circulating PCSK9 to LDLR at the PM is followed by endocytosis of the PCSK9-LDLR complex, a process dependent on the adaptor protein ARH [86]. In the endosomes, PCSK9 prevents the acid-dependent conformational change of LDLR necessary for its recycling to the PM, thereby stimulating degradation of the receptor in the LE/LY [88,89]. In addition to the inhibition of the extracellular binding of PCSK9 to LDLR through antibody-based therapies, blocking both the intracellular and the extracellular PCSK9-mediated LDLR degradation pathway by RNA-targeted therapy successfully lowers plasma LDL cholesterol and the risk of atherosclerotic cardiovascular disease [90,91].

Although the LDLR degradation pathways are well described, the mechanisms coordinating LDLR recycling were not understood until recently, when studies demonstrated that two protein complexes of the endosomal sorting machinery, CCC (COMMD/CCDC22/CCDC93) and WASH (Wiskott-Aldrich syndrome protein and SCAR homolog) [[92], [93], [94]], facilitate the recycling of LDLR back to the PM (Figure 2) [12,13]. The CCC complex consists of three core components, coiled-coil domain-containing (CCDC) CCDC22, CCDC93, and C16orf62 (recently renamed to VPS35L) and members of the Copper Metabolism MURR1 Domain-containing (COMMD) protein family [92]. Previous studies have shown that the CCC complex interacts with the WASH complex to maintain copper homeostasis by facilitating the endosomal trafficking of the copper-transporting proteins ATP7B and ATP7A [92,[95], [96], [97], [98], [99], [100]]. The WASH complex is composed of five subunits (WASHC1-C5) and activates the actin-related protein 2/3 (Arp2/3) complex to induce actin polymerization at endosomal sorting domains, which is required for endosomal receptor trafficking (Figure 2) [93,94]. Recently, the CCC complex has been shown to regulate the level and activity of WASH on the endosomal compartment to control endosomal protein recycling [101].

In mice and dogs, lack of one of the components of the CCC complex destabilizes the entire CCC core complex, resulting in reduced LDLR levels at the PM, enhanced lysosomal degradation of LDLR, impaired LDL cholesterol uptake, and ultimately increased plasma LDL cholesterol [13,102]. Furthermore, in ApoE3∗Leiden mice, a mouse model with a human-like lipoprotein profile, perturbation of the CCC complex exacerbates dyslipidemia and atherosclerosis [13].

In humans, hypomorphic mutations in *CCDC22* cause the severe developmental disorder X-linked intellectual disability (XLID) [103,104], and a homozygous splice site mutation in *WASHC5* causes Ritscher-Schinzel Syndrome 1 [105], both severe developmental disorders. XLID and Ritscher-Schinzel Syndrome 1 patients also suffer from hypercholesterolemia, illustrating that, across species, the endosomal recycling machinery is conserved to maintain cholesterol homeostasis [102]. Furthermore, a recent study revealed that a common genetic variant in *CCDC93* in humans is associated with reduced plasma LDL cholesterol levels, and correlated with lower risk of myocardial infarction and cardiovascular mortality [106]. The variant is associated with enhanced stability of the CCDC93 protein and probable improved functioning of the CCC complex, likely leading to better LDLR recycling and reduced plasma LDL cholesterol levels [106].

The WASH complex acts in concert with the CCC complex, and hepatic loss of the WASH components also results in increased plasma LDL cholesterol due to impaired endosomal trafficking of LDLR [14,102]. In addition to impaired recycling of the LDLR, hepatic WASH depletion also impairs endosomal recycling of LDLR-related protein 1 (LRP1) and scavenger receptor class B type I (SR-BI) [14]. LRP1 cooperates with LDLR in the uptake of remnant lipoproteins [107], while SR-BI mediates the uptake of CE from high-density lipoprotein particles [108]. Furthermore, it has been shown that the PCSK9-mediated LDLR degradation pathway does not rely on the endosomal LDLR trafficking itinerary facilitated by the WASH complex, whereas IDOL-dependent LDLR degradation does [14,109] (Figure 2). Taken together, the CCC/WASH axis seems to be essential for the endosomal trafficking of the lipoprotein receptors LDLR, LRP1, and SR-BI, and hence for hepatic uptake of cholesterol carried by different lipoproteins.

Mechanistically, it is well established that WASH is recruited to the endosomes by a trimeric protein complex called retromer, which is composed of VPS35, VPS26, and VPS29 [110,111] (Figure 2). Retromer is known to coordinate endosomal trafficking of a wide variety of cargo molecules, including Glut1, β2 adrenergic receptor, cation-independent mannose-6-phosphate receptor (CI-M6PR), and sortilin (see section 7) [[112], [113], [114], [115]]. However, a distinct retromer-independent trafficking pathway has recently been revealed by the identification of retriever, a retromer-like protein complex consisting of VPS35L (previously C16orf62), VPS26C (previously DSCR3) and VPS29 [116]. *In vitro* studies have shown that VPS35L does participate in the retriever as well as in the CCC complex [13,92,116] (Figure 2). Retriever localizes to the endosomes by binding to the CCC complex, and associates with both CCC and WASH to promote cargo retrieval and recycling [116]. Retriever may facilitate cargo recycling by coupling to sorting nexin SNX17 (Figure 2), an adaptor protein known to mediate sorting of proteins with NPxY/NxxY-sorting motifs in their intracellular cytosolic domains, such as LRP1 and LDLR [[116], [117], [118], [119]].

Although *in vitro* studies have implicated that retriever facilitates the recycling of members of the LDLR family and retromer SR-BI [113,116], it remains unclear whether these complexes are indeed required for the endosomal transport of these receptors *in vivo*. Likewise, it has not yet been determined whether a functional interaction takes place between the CCC/WASH axis and the adaptor protein phosphotyrosine interacting domain containing 1 (PID1). PID1 was recently shown to mediate hepatic uptake of lipoproteins by regulating the localization of LRP1 [15]. In summary, these findings indicate that the intracellular trafficking of lipoprotein receptors for hepatic cholesterol uptake is tightly regulated by several proteins, but how these are interconnected to maintain cholesterol homeostasis remains to be resolved.

In addition, it is important to note that altered cholesterol homeostasis changes membrane fluidity and domain formation, which can alter receptor trafficking and their downstream signaling pathways [8,120,121]. It is evident that changes in membrane cholesterol composition can directly modify the motility and functional properties of endolysosomal compartments, thereby affecting intracellular cargo trafficking and transport [[122], [123], [124]]. In addition, the positioning of LE/LY and formation of LE/LY-ER contacts is highly dependent on cholesterol levels [125]. This has also been observed in models of endolysosomal cholesterol accumulation, such as in NPC disease or other lysosomal storage diseases, which results in clustering of LE/LY and defects in vesicle trafficking [[126], [127], [128]]. Taken together, these data suggest that there is a bidirectional connection between cholesterol homeostasis and intracellular cargo trafficking, which indicate that intracellular cholesterol accumulation observed in metabolic diseases, such as in MALFD, might influence the functioning of the endolysosomal system.

## 7 Endosomal sorting proteins affect lipid metabolism beyond regulating cholesterol uptake

Mice with a hepatic deficiency of the CCC component COMMD1 are more prone to develop diet-induced hepatic steatosis [129]. The underlying mechanism is unclear, but depletion of the CCC-associated WASH complex results in decreased expression of genes regulated by the transcription factor liver X receptor (LXR) [14]. LXR regulates the expression of genes involved in fatty acid synthesis, as well as cholesterol efflux and excretion, and increased LXR activity is associated with hepatic steatosis [14,130]. Despite the reduced LXR activity, hepatic WASH-deficient chow-fed mice did not show differences in hepatic cholesterol and triglyceride levels [14].

It is furthermore interesting to mention that the WASH component WASHC2 (FAM21) is also localized to ER tubules [131]. Recent studies have shown that MCS between ER and endosomes are essential for the position and timing of WASH-associated endosomal fission events [93,131], yet there is no direct evidence for the role of WASH in the formation of endosome-ER MCS nor in cholesterol transport between these organelles.

It is known that MAFLD is associated with lysosomal dysfunction and impaired autophagy. MAFL patients show reduced expression of lysosomal acid hydrolases and lysosomal acid lipase (LAL), which hydrolyzes CE and TG [[132], [133], [134]]. Deficiency of LAL results in lysosomal cholesterol accumulation and induces dyslipidemia and steatosis [135]. Macroautophagy, the best studied form of autophagy (hereafter referred to as autophagy), is a lysosome-mediated degradation process for removal of intracellular material, such as organelles and protein aggregates, to provide energy during nutrient deprivation. Intracellular materials are sequestered by autophagosomes, which then fuse with lysosomes to form autolysosomes, where contents are degraded by lysosomal acid hydrolases (Figure 2) [136]. In 2009, the first link between autophagy and lipid metabolism was demonstrated by Singh et al., who reported that autophagy regulates breakdown of triglycerides (lipophagy) and may thus have anti-steatogenic properties [137]. Although some findings are still contradictory, possibly due to contextual differences between studies, the current consensus is that autophagy is reduced in MAFLD (reviewed by [138,139]). As autophagy is closely related to lysosomal function, it is therefore not surprising that conditions involving lysosomal cholesterol accumulation, such as NPC disease, have been associated with autophagic dysfunction. Lysosomal cholesterol accumulation increases the induction of autophagy, resulting in impaired autophagosome clearance and accumulation of autophagic vacuoles in NPC cells [[140], [141], [142]]. In addition, free cholesterol loading in HepG2 cells results in autolysosome accumulation and impaired clearance of autolysosome cargo [143]. This suggests that accumulation or mistrafficking of lipids modulates autophagic processes. On the other hand, inhibition of autophagy could decrease lysosomal cholesterol accumulation in NPC cells [142], which shows that the interaction between lipids and the autophagic pathway works both ways.

Interestingly, WASH deficiency in MEFs and mouse hepatocytes alters the architecture of the endolysosomal network [14,94,144], with smaller and more tubulated lysosomes [14]. These endolysosomal abnormalities are accompanied by increased autophagic flux in MEFs [145]. In addition to WASH, retromer has also been linked to lysosomal dysfunction and autophagy through its regulation of CI-M6PR and ATG9 trafficking [114,115,146]. CI-M6PR is one of the sorting receptors responsible for delivering acid hydrolases to LY [147]. These hydrolases are required for proper lysosomal function, and impaired delivery results in reduced lysosomal proteolytic capacity [148]. Mislocalization of CI-M6PR by aberrant retromer function in MEFs deficient in the small GTPase Arf6 was accompanied by free cholesterol accumulation in LE/LY [149]. This accumulation was associated with mislocalization of NPC2, likely due to mistrafficking of CI-M6PR [149], a known transporter of NPC2 [150]. In addition, the Parkinson's disease-related *VPS35* mutation is associated with impaired autophagy, likely by decreased recruitment of the WASH complex to the endosomes, as loss of WASH in HeLa cells also results in a defect in autophagy [151]. Although these data are contrary to the results observed in WASHC1-deficient MEFs [145], these findings clearly indicate a role for WASH and retromer in both lysosomal function and autophagy (Figure 1). However, hepatic WASHC1 deficiency in mice on a regular diet does not affect hepatic cholesterol or triglyceride content; thus whether hepatic WASH plays a role in autophagy, and subsequently in hepatic lipid accumulation, requires further investigation. Additional studies are also required to elucidate the contribution of retromer to lysosomal function and autophagy and, hence, to MAFLD.

Another known cargo of retromer is sortilin [112]. Sortilin, member of the vacuolar protein sorting 10 protein (Vps10p) domain family, is a lysosomal sorting receptor that delivers ligands mainly from the Golgi network to endolysosomal compartments and is subsequently transported back to the Golgi by retromer [112]. Sortilin is a known regulator of lipoprotein metabolism, as it is shown to regulate VLDL production and to be involved in LDL uptake, independent of LDLR (reviewed in [152]). However, some findings regarding the role of sortilin in lipid metabolism are contradictory, indicating that its action is rather complex [152,153]. Interestingly, several reports have demonstrated that deficiency of sortilin in mice with diet-induced obesity attenuates hepatic steatosis [154,155]. Mechanistically, this effect has been linked with reduced activity of acid sphingomyelinase (aSMase) [155]. In addition, sortilin-deficient mice fed a high-fat diet showed decreased free cholesterol accumulation due to reduced lysosomal targeting and degradation of carboxylesterase 1, an enzyme that catalyzes the breakdown of cholesterol esters in the liver [156]. Furthermore, loss of the hepatic (pro)renin receptor reduces sortilin-dependent LDL uptake [157]. Altogether, these data indicate that the retromer-cargo sortilin may be an important factor in MALFD and hepatic LDL cholesterol uptake.

## 8 Conclusions

*In vitro* studies have established that the endolysosomal network is essential for controlling cell homeostasis [9], but emerging evidence now shows that it is also crucial in maintaining metabolic homeostasis at the organismal level. Dysfunction of endolysosomal proteins can directly impair multiple steps of the endolysosomal pathways, thereby deregulating cellular and organismal homeostasis and contributing to disease development [158]. Recent studies have identified multiple endolysosomal proteins that regulate uptake, sorting, intracellular transport, and degradation of proteins and lipids. Some of these proteins have already been implicated in the pathogenesis of MAFLD, but the physiological role of most proteins is still not well understood. Further research is needed to enhance our understanding of their contribution to MAFLD and whether their functioning is affected by metabolic challenges [159].
